# The Weight and Height Percentiles in 6−18 Year Old Children in Kayseri and Comparison with Istanbul Data

**DOI:** 10.4008/jcrpe.v1i3.47

**Published:** 2009-02-03

**Authors:** Nihal Hatipoğlu, Selim Kurtoğlu, Ahmet Öztürk, Mümtaz Mustafa Mazıcıoğlu

**Affiliations:** 1 Erciyes University, Faculty of Medicine, Department of Pediatrics, Division of Pediatric Endocrinology & Metabolism, Kayseri, Turkey; +90−352 438 00 76+90−352 437 58 25selimk@erciyes.edu.trErciyes Üniversitesi, Tıp Fakültesi, Çocuk Sağlığı ve Hastalıkları AD, Neonatoloji ve Pediatrik Endokrinoloji ve Metabolizma Bölümü−Kayseri−Turkey

**Keywords:** weight, height, percentile curve

## Abstract

**Background**: One of the mostly used and preferred method in following the growth of children is to plot weight and height values of the children on standard percentile charts. It is essential for each country to use its own populations’ updated percentile curves. However, data on the growth of children living in different regions are also needed for comparison with the national standards.

**Methods**: This study was conducted in Kayseri with a trained team in order to obtain anthropometrical measurements in children and adolescents. Weight and height measurements from 5727 (2785 boys, 2942 girls) healthy school children (aged between 6 to 18 years) from all socioeconomic levels were randomly selected. Smoothed percentile curves were produced by LMS method.

**Results**: Smoothed percentile curves including the percentile values for 3^rd^, 5^th^, 10^th^, 25^th^, 50^th^, 75^th^, 85^th^, 90^th^, 95^th^ and 97^th^ and standard deviation scores were calculated for boys and girls. The 3^rd^, 50^th^ and 97^th^ centiles of weight and height of these children were compared with the respective values of the established national standards obtained from Istanbul children.

**Conclusions**: This study presents data and smoothed percentile curves for weight and height measurement of healthy central Anatolia children aged 6 to 18 years. Nationwide studies are needed to bring out the regional differences in our country.

**Conflict of interest:**None declared.

## INTRODUCTION

Through basic anthropometric measurements such as height and weight, the growth patterns of children can be evaluated and considerable information can be achieved about their nutritional status and global health. In the last approximately three decades, reference curves recommended by World Health Organization (WHO) have been used to evaluate nutritional status of children in the world.(1, 2) However, a child’s growth can demonstrate differences due to environmental, genetic and nutritional factors.(3) Growth patterns demonstrate differences among different countries and among populations of different ethnic origin.(4, 5) These differences in growth patterns have been reported to persist even after controlling for various factors such as nutrition, environment, maternal care, child care, income distribution, gross domestic product, health services and political influences.(5, 6) It is therefore recommended that every country should use reference height and weight curves based on measurements on their own children. 

There are few studies on the growth of school children in Turkey.(7, 8, 9, 10, 11, 12) All of these studies with one exception(11) are based on measurements of children residing in Istanbul. The recent study by Neyzi et al (12) was undertaken to update the growth reference charts for Turkish children. These new charts are based on measurements of Istanbul children from well−to−do families.(12)

In this paper we present the reference data on height and weight for Turkish subjects aged 6−18 years living in a large city in Turkey, collected in 2006. Height and weight references were compared with available references of Turkish children living in Istanbul.(12)

## MATERIALS AND METHODS

This is a cross−sectional study conducted in the primary and secondary school children living in Kayseri. The city, situated in the central Anatolia of Turkey, has more than 1,000,000 inhabitants and is a leading industrial and trade center which receives emigrants from other parts of Turkey for employment. The data for this present analysis were obtained from the study entitled “Determination of Anthropometric Measurements of Turkish Children an Adolescents (DAMTCA)” conducted between February and April 2006. The Erciyes University School of Medicine Ethics Committee and the Kayseri province Education Board approved the study protocol.

This study used a stratified multistage probability sampling designed for school children. Firstly, the schools were selected randomly by a stratified sampling method according to socio−economic levels from among state and private schools, which represent the city centre and rural districts, A total of 47 (23 primary, 24 secondary) schools were selected randomly from among 699 schools in the Kayseri province. Thus, sampling was carried out from every area and included cases from all socio−economic levels. Secondly, random sampling of children and adolescents aged 6 to 18 years was done from schools enrollment. A total of 5727 primary and secondary school students (2942 girls, 2785 boys) were selected for the study. The chronological age was calculated as the decimal age from birth date and date of examination.

The excluding criteria from study were defined as the presence of any disorder affecting growth such as a metabolic, neurological or gastrointestinal condition, and use of any kind of medication. Parents’ written consent was obtained prior to the study, and the procedures followed were in accordance with those outlined by the Declaration of Helsinki.

Trained health technicians performed all measurements. Weight and height measurements were repeated twice and the mean value of the two measurements was used. Weights were measured using a standard beam balance sensitive to 0.1 kg (Tefal Ultraslim, France) with the children in their underclothes and without shoes. Height was determined to the nearest 1 mm using a Seca model portable stadiometer. The portable scales and stadiometers were calibrated daily.

**Statistical analysis**

Construction of the percentile curves was performed with the LMS Chart Maker Pro version 2.3 software program (The Institute of Child Health, London), which fits smooth percentile curves to reference data using the LMS method. The smoothed percentile curves for body mass index (BMI) were constructed by the LMS method.(13) This method summarizes percentiles at each age based on the power of age−specific Box−Cox power transformations that are used to normalize data. These three quantities depend on age. The final curves of percentiles are produced by three smooth curves representing L (Lambda; skewness), M (Mu; median) and S (Sigma; coefficient of variation). Percentile values were measured by the LMS Chart Maker Pro version 2.3 software programs, and percentile curves (3^rd^, 5^th^, 10^th^, 25^th^, 50^th^, 75^th^, 85^th^, 90^th^, 95^th^ and 97^th^) were constructed by Microsoft Office Excel® version 2003. Descriptive statistics for each whole year (e.g., 6.00−7.99 y, etc.) for each sex were calculated by SPSS version 13.0 (Illinois, Chicago, USA).

## RESULTS

Tables 1 and 2 show smoothed percentiles of 3^rd^, 5^th^, 10^th^, 25^th^, 50^th^, 75^th^, 85^th^, 90^th^, 95^th^ and 97^th^ in girls and boys and Table 3 presents the LMS curves and standard deviation scores for weight and height by age and sex. Figures 1 and 2 compare the 3^rd^, 50^th^ and 97^th^ centiles of weight and height for Turkish girls and boys of Istanbul and Kayseri.(12)

**Figures 1:**
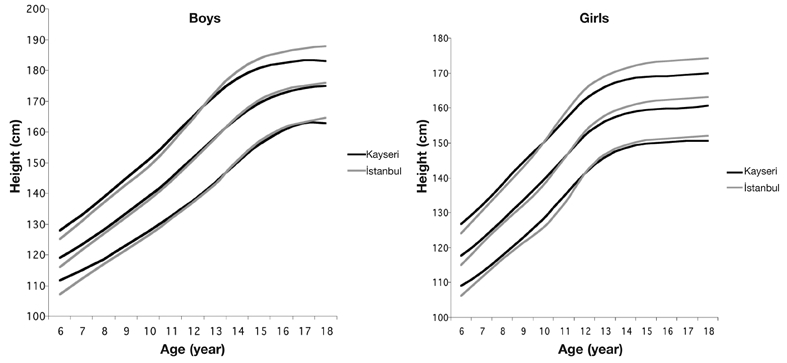
Comparison of 3^rd^, 50^th^ and 97^th^ centiles of height distribution in boys (left) and girls (right) between Kayseri and Istanbul.

**2:**
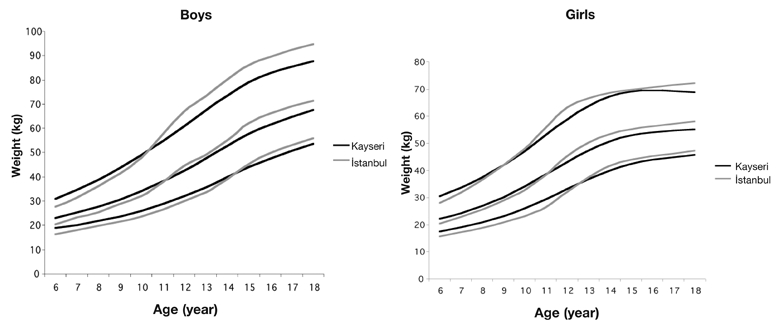
Comparison of 3^rd^, 50^th^ and 97^th^ centiles of weight distribution in boys (left) and girls (right) between Kayseri and Istanbul.

**Tables 1:**
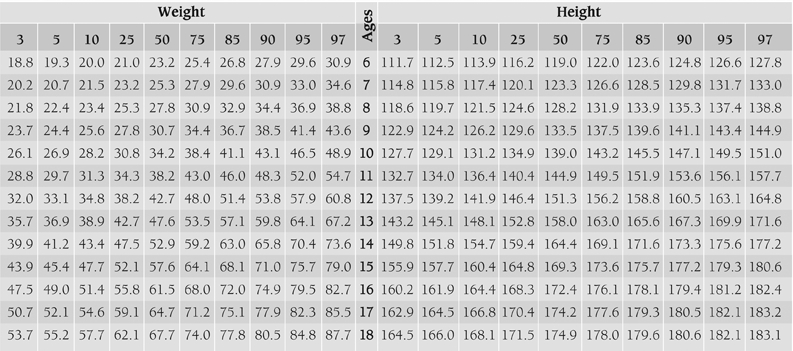
The smoothed age−specific weight and height percentiles for the boys.

**2:**
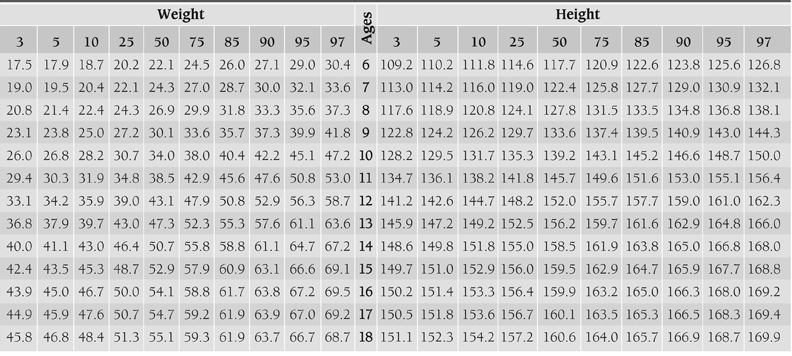
The smoothed age−specific weight and height percentiles for the girls.

**Table 3:**
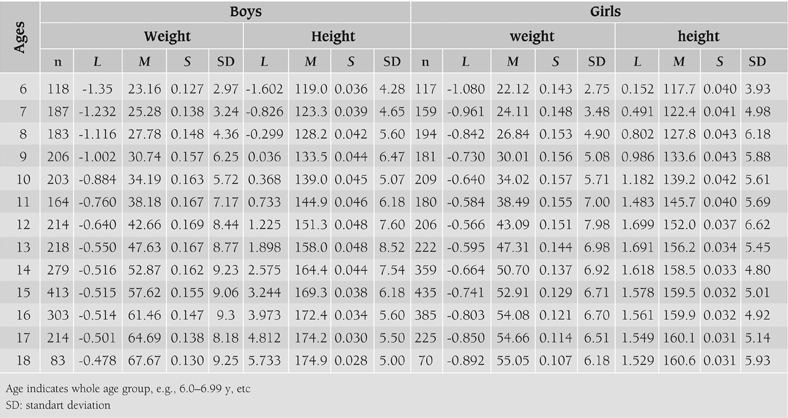
LMS and standard deviation values for weight and height by age and sex.

## DISCUSSION

The reference percentile curves for height and weight of Turkish children have first been established by Neyzi et al. in 1970s and have been used since then.(9) The same group of investigators have recently published updated reference curves for Turkish children of 6−18 years.(12) Both studies have enrolled schoolboys and girls of higher socioeconomic state who lived in Istanbul and have presented the ideal levels of society as “predictive” reference values. Istanbul is the largest city which receive migration from all of Turkey and 17% of Turkish population reside in Istanbul.

Our study has been conducted in school age children who live in Kayseri, the third biggest city of Central Anatolia and which receives migration from other regions in accordance with the industrialization process that has gained velocity for the last 10 years and that could reflect characteristics of Turkish children like Istanbul. A mixture of all socioeconomic levels have been included in Kayseri study, while in the study conducted by Neyzi et al. children of a high socioeconomic status have been enrolled.(12) Thus, reflection of current social values instead of ideal social target has been the aim in this study to be demonstrated. Thus in two different cities of Turkey, based on two different studies, the effects of environment and socioeconomic aspects on societies sharing the same ethnic origin have been reviewed and compared.

While height values of boys in Kayseri region are 1.4 cm higher in average than Istanbul group between 6 and 12 years, it was observed that heights of children from Istanbul are 0.89 cm higher between 14 and 18 years. As for girls, while children of Kayseri are 1.55 cm taller in average than Istanbul group in the first 12 years of life, it is seen that starting from age 12, girls of Istanbul group are 2.03 cm taller in average.

Body weight values are 2.3 kg heavier in male children of Kayseri region up to 10 years of age, while it has been found that an average of 3.3 kg is gained in children of Istanbul group from 11 years of age onwards that increases in parallel with growing age. Similarly, while girls of Kayseri region are 1.4 kg heavier in average up to 10 years of age, from age 12, the girls that live in Istanbul were found to be 2.5 kg heavier in average in an increasing fashion.

When 3^rd^ and 97^th^ percentile values of children of Kayseri and Istanbul groups are compared, the emphasis being especially on 97th percentile, a significant difference is observed between two groups after puberty (Figure 1, 2). We can say that this difference should be taken into account especially while evaluating children who show deviations from normal values.

For both sexes, while height and weight values are greater in prepubertal children in Kayseri region, the values tend to lag behind in the pubertal period compared to the children that live in Istanbul. The reason for this observation could be explained by environmental and economical factors. Kayseri has been involved in a rapid industrialization process in the last 10−12 years and a rapid increase in socioeconomic level has been observed. The increase in income through this 10−12 years process has shown its effects on weight and height improvements of prepubertal children. As reflections of this surge could not yet be seen in pubertal period, we conclude that the values of weight and height might have stayed behind the values of children in Istanbul for this reason. The increment in weight and height during pubertal period can be observed in studies that are to be conducted in Kayseri in the future if our hypothesis is true.

In addition, it might be that different nutritional habits and more importantly limited physical activity could affect Istanbul pubertal group. In a similar study, weight and height reference values of children in Northern and Central regions of Italy have been compared with values of children in Southern Italy and it has been shown that there might have been differences between regions that might derive from economic and environmental factors. (14, 15)

However, some of the differences observed in these two regions may be explained with the differences in the methodologies of these studies. While Istanbul study has been conducted by longitudinal follow up of the same cohort of children, Kayseri study has been conducted cross−sectionally. This is one of the drawbacks of the present study.

The percentiles presented in this study may not be recommended as national standards because according to WHO, standards should be prepared from the well−to−do children of the society. However the aim of this study was to reflect the regional differences in the society due to ethnic or environemental factors.

As a result, we may conclude that the percentile values established in boys and girls of 6−18 age groups from Kayseri can be utilized while evaluating growth of children living in all socioeconomic levels in our country. However, it should be emphasized that new values have to be obtained and updated in further studies performed at specific intervals.

**Figure 1:**
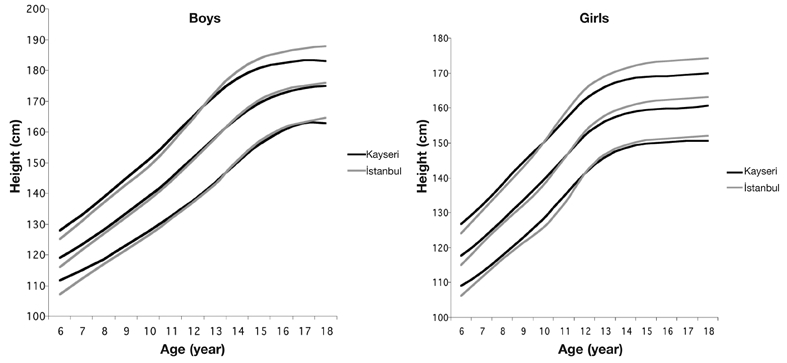
Comparison of 3^rd^, 50^th^ and 97^th^ centiles of height distribution in boys (left) and girls (right) between Kayseri and Istanbul.

**2:**
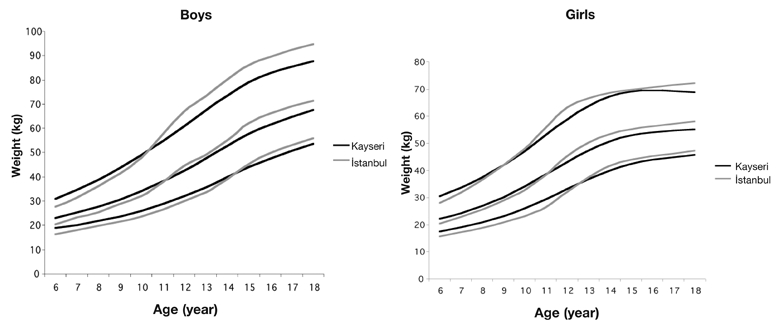
Comparison of 3^rd^, 50^th^ and 97^th^ centiles of weight distribution in boys (left) and girls (right) between Kayseri and Istanbul.
